# Retrieval of a Dislodged Catheter Using Combined Fluoroscopy and Intracardiac Echocardiography

**DOI:** 10.1155/2015/610362

**Published:** 2015-05-13

**Authors:** Gus Mitsopoulos, Robert F. Hanna, Sidney Z. Brejt, Greg E. Goldstein, Vladimir Sheynzon, Joshua L. Weintraub, William A. Gray

**Affiliations:** ^1^Department of Vascular & Interventional Radiology, Columbia University, 622 West 168th Street, P.O. Box 1-301, New York, NY 10032, USA; ^2^Department of Cardiology, Columbia University, 622 West 168th Street, P.O. Box 1-301, New York, NY 10032, USA

## Abstract

This report details a method of percutaneous, transluminal retrieval of an intracardiac foreign body using fluoroscopy in combination with intracardiac echocardiography. During retrieval, intracardiac echocardiography (ICE) provided real-time anatomic localization of a constantly moving, almost radiolucent micropuncture coaxial dilator fragment with respect to the tricuspid and pulmonary valves. This method may serve as a crucial aid in retrieval of intracardiac foreign bodies that are difficult to see with fluoroscopy and which may be adjacent to cardiac valves.

## 1. Background

Intracardiac foreign bodies and their removal have been described in prior case reports. Catheter fragments, pacemaker electrodes, needles, and stents are the most common [1]. The first description of embolization of a catheter fragment was made in 1954 [2]. The first percutaneous, transluminal recovery of an intravascular foreign body can be credited to Porstmann et al., 1967, in connection with their catheter technique for patent ductus arteriosus (PDA) closure [3]. Before that, techniques for removal of intracardiac foreign bodies included thoracotomy and transluminal retrieval via saphenous vein cutdown [4].

Although it has been reported that foreign bodies have remained in the body for up to seventeen years without major complications, there are many well-known major complications of foreign bodies, such as sepsis, endocarditis, arrhythmias, valvular malfunction, cardiac perforation, pulmonary embolism, and death [5], with an overall 71% incidence of death or major complication [6]. In terms of location with respect to the heart, mortality is highest with the embolized fragment located in the right atrium (RA)/right ventricle (RV), slightly lower in the vena cava, and lowest in the pulmonary artery [5].

Retrieval of intracardiac foreign bodies, similar to techniques used for intravascular foreign bodies, hinges mostly on the use of snare devices and forceps. Complications resulting from the removal of foreign bodies are rare, and the technical success rate has been reported to reach up to 100% [7]; however, caution is advised when the foreign body is adjacent to a valve. These procedures have historically been performed with fluoroscopy as the sole imaging modality. This case report demonstrates the successful use of intracardiac echocardiography as the dominant imaging method to visualize and help guide retrieval of an intracardiac foreign body. Given the educational purpose of our case report, IRB approval was not obtained.

## 2. Case

47-year-old female with past medical history of hyperthyroidism and remote history of right lower extremity deep venous thrombosis (DVT) during pregnancy 10 years ago resulting in a postphlebitic syndrome consulted an interventional radiologist at an outside free-standing clinic. Her DVT resolved with anticoagulation and she was otherwise well, taking only methimazole and propranolol for her hyperthyroidism, and had had no past surgeries. However, her symptoms of right leg pain, heaviness, and swelling had progressed since her initial DVT. Given her worsened symptoms of superficial venous insufficiency, she was scheduled for endovenous laser therapy (EVLT) of the right great saphenous vein.

During distal greater saphenous venous access for the procedure with a micropuncture kit, the inner 3 F dilator of the micropuncture and 0.018 wire were removed; however, the shaft of the dilator appeared to have fractured and was retained intravenously. Shortly after, the patient began complaining of chest pain and the electrocardiography (EKG) monitor revealed multiple episodes of premature ventricular contractions (PVCs). Emergency medical services (EMS) were activated and she was transported to our institution.

On presentation, the patient complained of a “throbbing” left upper chest pain (8/10 in severity), radiating to left axilla, with mild associated dyspnea and dizziness. Her pain was worse with deep inspiration and movement. She denied a cardiac history and said she never had pain like this before. She also denied syncope, nausea, vomiting, or diaphoresis. On physical exam, she was uncomfortable but alert and oriented. Her vital signs were significant for bradycardia but otherwise unremarkable (blood pressure of 134/77 mm Hg, heart rate 50 beats per minute, respiratory rate 18 breaths per minute, and temperature 97.6 degrees Fahrenheit). Her cardiac, respiratory, and neurologic exams were normal. EKG in the emergency department (ED) showed sinus bradycardia and incomplete right bundle branch block (RBBB). An immediate troponin level was elevated at 0.09 ng/mL (normal is less than 0.03 ng/mL), increasing further several hours later to 0.14 ng/mL.

**Figure 1:**
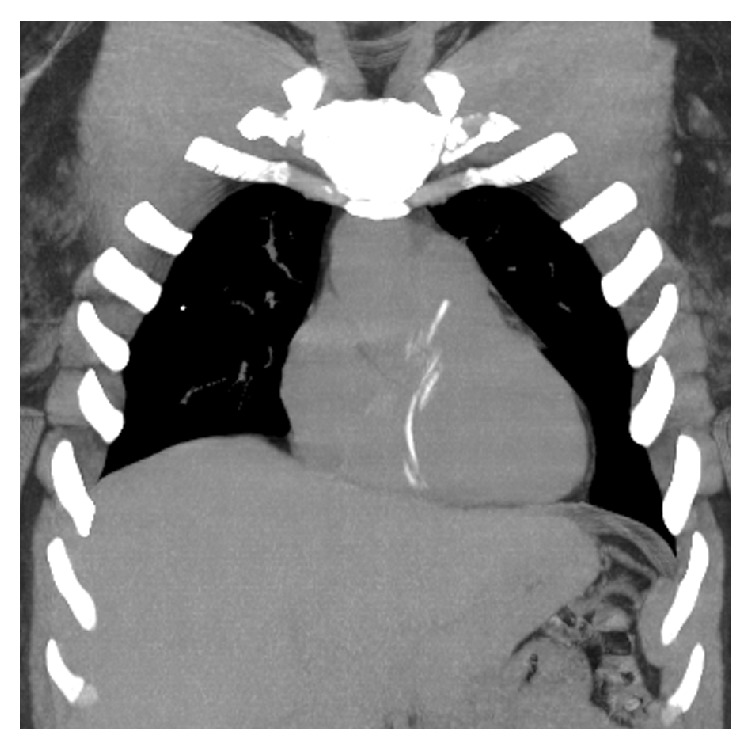
Coronal reformat noncontrast CT MIP image demonstrates the dislodged catheter extending from the right atrium through the right ventricle terminating in the pulmonary outflow tract.

Given the high suspicion of an intracardiac location of the retained catheter, imaging was ordered to locate the missing foreign body, beginning with plain films of the chest, abdomen, and right lower extremity. These were negative for evidence of a radiopaque foreign body. A transthoracic echocardiogram was also ordered, which demonstrated mild tricuspid regurgitation but no visible foreign body (although the pulmonic valve and pulmonary artery could not be visualized due to patient body habitus). Finally, computed tomography (CT) images of the chest demonstrated a thin, curvilinear density representing the 10 cm × 1 mm fragment of the 3 F inner dilator extending from the right ventricle to the pulmonary outflow tract (Figure 1). The patient was then admitted for further work-up and telemetry monitoring.

While on telemetry, the patient had occasional PVCs, one episode of nonsustained ventricular tachycardia (NSVT) up to 5 beats, and ventricular bigeminy. Her chest pain symptoms persisted and were treated with intravenous morphine injections as needed.

The interventional radiology and cardiothoracic services were consulted for removal of the intracardiac foreign body. Given the patient's chest pain and arrhythmia as a result of the dilator fragment, a transcatheter removal was recommended and performed without delay.

**Figure 2:**
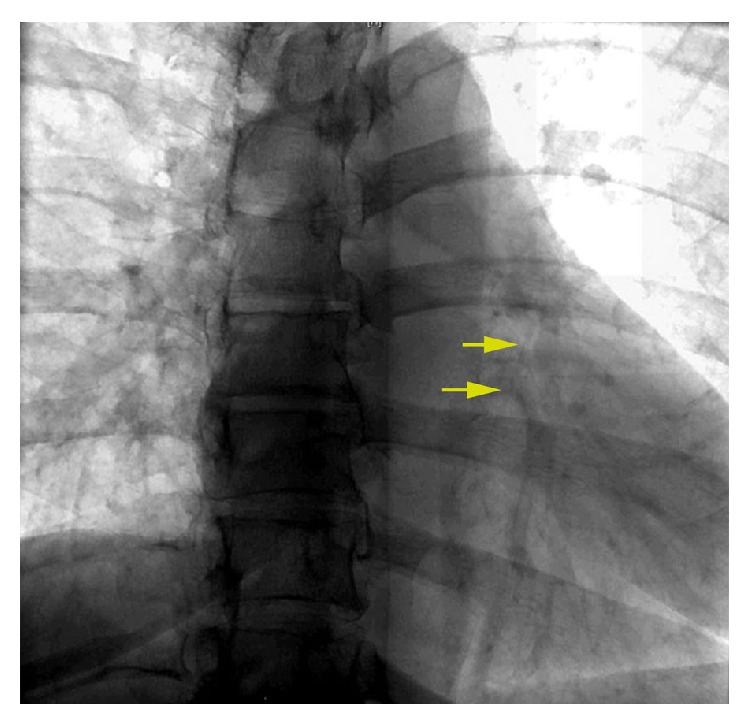
Fluoroscopy at the time of retrieval. Coned view of the heart faintly visualizes the dislodged catheter (arrow).

The patient was brought into the fluoroscopy suite and due to recent ventricular tachycardia documented on telemetry, defibrillator pads were prophylactically placed on the patient's chest and rhythm was monitored using the defibrillator unit. The initial chest X-ray was reviewed and a curvilinear density was localized in the region of the right ventricle. Fluoroscopy (Siemens Artis Zee, Munich, Germany) of the heart was performed to visualize the dilator fragment seen on chest X-ray and CT; however, the fragment was not initially visible using this method. A high-resolution cine-angiographic image of the cardiac silhouette was then obtained, which demonstrated a faintly visible long and thin tubular structure compatible with the dilator fragment (Figure 2). Having localized the fragment in this way, the interventionalist could better identify it on fluoroscopic imaging.

Access was obtained in the right common femoral vein which, given patient body habitus, was performed with a micropuncture kit. This was then exchanged for a long 12 F guiding sheath. A 15 mm ONE snare (single-loop) (Merit Medical, South Jordan, UT) was then advanced over the area where the foreign body appeared to be positioned on prior imaging. Using fluoroscopy, multiple attempts were made to snare the foreign body but these were unsuccessful. Additional attempts with a 12–20 mm EN snare (triple-loop) (Merit Medical, South Jordan, UT) did not prove more fruitful. These initial attempts at retrieval were difficult due to insufficient visualization of the foreign body during retrieval, the uncertain relationship of the foreign body to the tricuspid and pulmonic valves, and the inability to assess the location of the fragment from a three-dimensional perspective.

**Figure 3:**
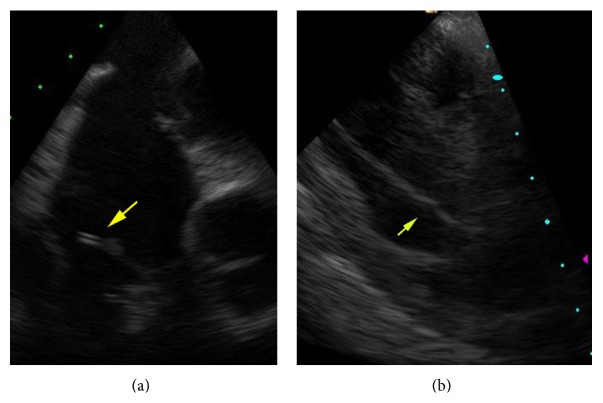
(a) Intracardiac ultrasound-dislodged catheter (arrow) is extending across the tricuspid valve. (b) Intracardiac ultrasound-dislodged catheter (arrow) is seen in the right ventricle.

The interventional cardiology service was then contacted to assist in the retrieval with the use of intracardiac echocardiography (ICE). A second puncture was made in the right common femoral vein 1 cm below the original sheath and a second (8 F) sheath was placed. An 8 Fr ICE probe (Siemens ACUSON AcuNav ultrasound catheter, Munich, Germany) was inserted and advanced to the low right atrium. Echo images demonstrated no pericardial effusion and the dilator fragment to be primarily within the right ventricle with a small portion of it extending across the tricuspid valve into the right atrium (Figure 3(a)). A 6 F 100 cm Judkins Right (JR4) guiding catheter (Cordis Corp., Warren, NJ) was then inserted through the 12 F sheath and used to direct the snare to the dilator fragment. Initial attempts to retrieve the proximal end of the fragment under ICE guidance were unsuccessful and made difficult by the location of the catheter to the tricuspid valve and associated subvalvular chordal apparatus, which tended to deflect the snare in unpredictable ways. Ultimately, attempts to snare the proximal end of the fragment resulted in it being displaced completely into the right ventricle (Figure 3(b)), requiring a repositioning of the ICE catheter into the right ventricle for more complete fragment visualization. The fragment was found to be completely contained within the right ventricle and did not extend across the pulmonic valve. Using this information, the snare was repositioned in the right ventricular outflow tract above the most distal extent of the fragment. The snare was then unsheathed and gently pulled back and over the distal tip of the fragment where it was successfully captured in its midsegment. The catheter was then removed through the 12 F sheath in the right groin, and its length matched the expected length thus confirming the likelihood of complete foreign body removal. The echo probe and sheaths were then removed and hemostasis at both sites was achieved with manual compression. The patient tolerated the procedure well.

The patient had immediate improvement of her chest pain upon removal of the dilator fragment. Telemetry monitoring overnight showed few PVCs, significantly decreased from prior. She was subsequently discharged home in good condition.

## 3. Discussion

Major complications of EVLT are rare [8]. However, in this case, a portion of the inner micropuncture 3 Fr dilator broke from its hub and embolized to the heart. Once the diagnosis of an intracardiac foreign body is made, the main question to be answered is whether it should be removed. As with any other complex scenario, this decision should be made on a case by case basis, weighing the risks of harm from the indwelling foreign body versus the risks undertaken when attempting to remove it. Discussion should also be undertaken with cardiology, cardiothoracic surgery, and interventional radiology as was done in this case.

It is generally agreed upon the fact that retrieval of intracardiac foreign bodies should be considered when size exceeds 5 to 10 mm, when shape is irregular, or when patient is symptomatic [9–12]. If the patient is asymptomatic and the above characteristics are absent, conservative management can be used, with close follow-up, anticoagulation to prevent thrombosis, and antibiotic prophylaxis against endocarditis [8, 9, 12].

Removal of our patient's intracardiac foreign body was indicated for multiple reasons: the dilator fragment was 10 cm in length and the patient was symptomatic with chest pain and arrhythmia after the fragment was noted to be missing. Difficulty was encountered in the fluoroscopy suite due to near radiolucency of the dilator and 3D representation in space. In fact, until this point, our fluoroscopy time was 47 minutes, nearly two-thirds of our case total of 70.2 minutes. Therefore, we enlisted the assistance of the interventional cardiology service, experienced in using ICE, in order to provide visualization of the foreign body as well as guidance for subsequent retrieval. Preoperative transthoracic/transesophageal echocardiography and even intraoperative epicardial echocardiography have been used to assist in the removal of intracardiac foreign bodies [13, 14].

In conclusion, this present case demonstrates endovascular retrieval of a 3 F dilator fragment, which was ultimately successful using both fluoroscopy and ICE for imaging guidance. Given difficulty in visualization of the foreign body and proximity to the valve, the use of ICE proved very helpful in retrieval.
